# Specific Uptake in the Bone Marrow Causes High Absorbed Red Marrow Doses During [^177^Lu]Lu-DOTATATE Treatment

**DOI:** 10.2967/jnumed.123.265484

**Published:** 2023-09

**Authors:** Jens Hemmingsson, Johanna Svensson, Andreas Hallqvist, Katja Smits, Viktor Johanson, Peter Bernhardt

**Affiliations:** 1 Department of Medical Radiation Sciences, Institute of Clinical Sciences, Sahlgrenska Academy, Gothenburg, Sweden;; 2 Department of Oncology, Institute of Clinical Sciences, Sahlgrenska Academy, Gothenburg, Sweden;; 3 Department of Oncology, Sahlgrenska University Hospital, Gothenburg, Sweden;; 4 Department of Surgery, Institute of Clinical Sciences, Sahlgrenska Academy, Gothenburg, Sweden; and; 5 Department of Medical Physics and Medical Bioengineering, Sahlgrenska University Hospital, Gothenburg, Sweden

**Keywords:** dosimetry, red marrow, ^177^Lu, SPECT, DOTATATE

## Abstract

Bone marrow suppression is a common side effect after [^177^Lu]Lu-DOTATATE treatment of neuroendocrine neoplasms. Neuroendocrine neoplasms share expression of somatostatin receptor type 2 with CD34-positive hematopoietic progenitor cells, potentially leading to active uptake in the radiosensitive red marrow region where these cells are located. This study aimed to identify and quantify specific red marrow uptake using SPECT/CT images collected after the first treatment cycle. **Methods:** Seventeen patients diagnosed with neuroendocrine neoplasms were treated with [^177^Lu]Lu-DOTATATE. Seven of them had confirmed bone metastases. After the first treatment cycle, each patient went through 4 SPECT/CT imaging sessions 4, 24, 48, and 168 h after administration. Monte Carlo–based reconstructions were used to quantify activity concentrations in tumors and multiple skeletal sites presumed to house red marrow: the T9–L5 vertebrae and the ilium portion of the hip bones. The activity concentration from the descending aorta was used as input in a compartment model intended to establish a pure red marrow biodistribution by separating the nonspecific blood-based contribution from the specific activity concentration in red marrow. The biodistributions from the compartment model were used to perform red marrow dosimetry at each skeletal site. **Results:** Increased uptake of [^177^Lu]Lu-DOTATATE was observed in the T9–L5 vertebrae and hip bones in all 17 patients compared with activity concentrations in the aorta. The mean specific red marrow uptake was 49% (range, 0%–93%) higher than the nonspecific uptake. The median (±SD) total absorbed dose to the red marrow was 0.056 ± 0.023 Gy/GBq and 0.043 ± 0.022 Gy/GBq for the mean of all vertebrae and hip bones, respectively. The patients with bone metastases had an absorbed dose of 0.085 ± 0.046 Gy/GBq and 0.069 ± 0.033 Gy/GBq for the vertebrae and hip bones, respectively. The red marrow elimination phase was statistically slower in patients with fast tumor elimination, which is in line with transferrin transport of ^177^Lu back to the red marrow. **Conclusion:** Our results suggest that specific red marrow uptake of [^177^Lu]Lu-DOTATATE is in line with observations of somatostatin receptor type 2–expressing hematopoietic progenitor cells within the bone marrow. Blood-based dosimetry methods fail to account for the prolonged elimination of specific uptake and underestimate the absorbed dose to red marrow.

## Visual Abstract

**Figure:**
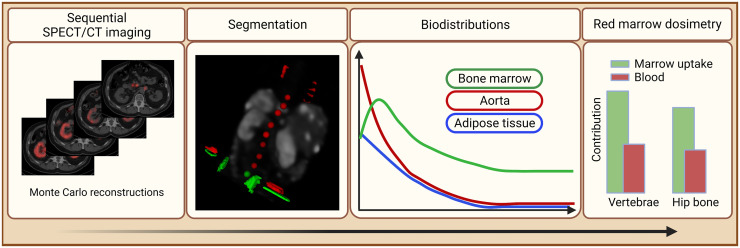

The effects on the bone marrow of treating neuroendocrine neoplasms with ^177^Lu-labeled DOTA^0^-Tyr^3^-octreotate ([^177^Lu]Lu-DOTATATE) are usually mild and transient, but up to 10%–15% of patients develop grade 3 or 4 hematologic toxicities that can be long-lasting and hamper subsequent therapies (1–3). Treatment effects on the red marrow are measured indirectly through blood sampling, but neither blood-based nor image-based dosimetry is routinely performed for the bone marrow.

Red marrow dosimetry is less straightforward than dosimetry for the kidneys, the other main dose-limiting risk organ, and suffers from limited accuracy, as the red marrow is heterogeneously distributed throughout cavities in trabecular bone (4). Furthermore, the comparatively low activity concentrations in the spongy bone require sufficiently long acquisition times for SPECT imaging. Red marrow is found throughout the skeleton; the largest quantities in adults are observed in the hip bones and lumbar and thoracic vertebrae (5). The size of the bone cavities in these skeletal regions and their location in the SPECT images make them suitable for image-based dosimetry. Compared with blood-based dosimetry, image-based methods can estimate the activity concentration from a section of spongy bone. However, similar to the blood-based approach, the specific activity distribution within the spongy bone, a region consisting mainly of trabecular bone, red marrow, marrow adipocytes (yellow marrow), and blood vessels, has to be assumed (6). Nevertheless, several image-based studies have demonstrated dose–response correlations, both for [^177^Lu]Lu-DOTATATE and for ^90^Y DOTA-d-Phe^1^-Tyr^3^-octreotide ([^90^Y]Y-DOTATOC), but these correlations are still too weak to be useful predictors of toxicity (7–9).

With blood-based red marrow dosimetry, the biodistribution of [^177^Lu]Lu-DOTATATE in red marrow is assumed to be similar to the biodistribution in blood. In a large prospective study by Garske et al., blood-based red marrow dosimetry resulted in low individual absorbed doses (population mean, 0.016 Gy/Bq), with no correlation to red marrow toxicity (10). However, Oomen et al. identified somatostatin receptor subtype 2 expression in CD34-positive immature progenitor cells from the red marrow, which may partly explain the higher absorbed doses obtained with image-based dosimetry (Table 1) (11).

**TABLE 1.** Studies Showing Mean Absorbed Dose (Gy/GBq) to Red Marrow Using Image-Based Dosimetry Methods

Study	Patients (*n*)	Red marrow	SD
Santoro (36)	9	0.043	0.019
Marin (37)	47	0.028	0.010
Del Prete (8)	34	0.038	0.024
Hagmarker (7)	24*	0.051	0.015
Huizing (34)	10	0.087	0.030
Kim (38)	20	0.065	0.061
Vergnaud (39)	13	0.040	0.030
Kamaldeep (40)	60	0.040	0.020

* Without bone metastases.

The objective of this study was to investigate whether any specific red marrow uptake can be quantified in sequential SPECT/CT images after [^177^Lu]Lu-DOTATATE treatments. A compartment model was applied to the image-based activity concentrations to remove the blood component based on data from the descending aorta, tumors, and the red marrow compartments in the lumbar and thoracic vertebrae and the ilium part of the hip bones (hereafter referred to as the hip bones). Data from the compartment model were then used to determine biodistributions for the tumors and red marrow compartments and to estimate the contribution of red marrow uptake to the absorbed dose to red marrow.

## MATERIALS AND METHODS

Seventeen patients diagnosed with neuroendocrine neoplasms were treated with [^177^Lu]Lu-DOTATATE (Lutathera; AAA) according to the recommendation of 4 cycles of 7.4 GBq/cycle. The median patient age was 77 y (range, 58–88 y), and 7 patients were confirmed to have bone metastases on pretherapeutic [^68^Ga]Ga-DOTATATE PET/CT. This retrospective clinical study was approved by the Swedish Ethics Review Board (diarienummer 2020-05092), and the requirement to obtain informed consent was waived.

### Image Acquisition

Four SPECT/CT images were acquired after the first treatment cycle at 4.1 ± 1.1 h, 18.8 ± 2.4 h, 53.0 ± 21.8 h, and 169.6 ± 10.6 h (mean ± SD) after injection. Two Tandem Discovery 670 Pro SPECT/CT cameras (GE Healthcare) with medium-energy general-purpose collimators were used to collect 120 (60 × 2) projections (30 s per projection) with the energy window set at 208.4 keV ± 10% and 2 scattering windows contiguous with the emission window, above and below, with widths set to 5% of the photopeak. SPECT images were reconstructed using the Monte Carlo–based ordered-subset expectation maximization (OSEM) Sahlgrenska Academy reconstruction code to obtain attenuation, scatter, and collimator–detector response corrections (12). The Monte Carlo OSEM reconstruction parameters were set to 6 iterations and 10 subsets, and backprojection was performed with a point-spread beam for reduced noise.

### Image Analysis

**FIGURE 1.**
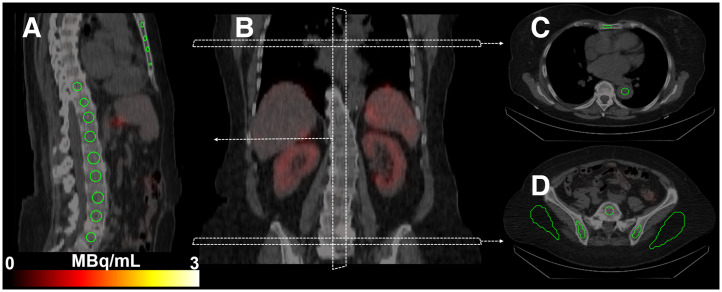
SPECT/CT images from patient 14 show delineated VOIs (green contours). (A) Sagittal view of delineated T9–L5 vertebrae and sternum. (B) Orientation and position of each image slice in coronal plane. (C) Transverse slice of descending aorta and sternum. (D) Hypogastric and iliac regions and delineations of L5 vertebrae, left and right hip bones, and subcutaneous adipose tissue.

Multiple volumes of interest (VOIs) were manually drawn on the SPECT/CT images for each time point using an in-house–developed image platform (12). First, the spongy bone component of 2 anatomic regions presumed to house significant quantities of red marrow was delineated: the T9–L5 thoracic and lumbar vertebrae and the hip bones (Fig. 1). The other main constituents in these regions were assumed to be yellow marrow, trabecular bone, and blood vessels (6). The VOIs within the vertebrae were delineated with a centrally placed 4-mL sphere unless metastases were present, in which case they were delineated manually. The VOIs within the left and right hip bones were manually delineated. A VOI within the descending thoracic aorta was delineated to represent a blood compartment similar to the method commonly applied in PET imaging to determine activity concentrations in the blood (13). To represent tissues with both specific and nonspecific activity uptake, VOIs within tumor tissue and subcutaneous adipose tissue were delineated. Extracted voxel values from the VOIs were translated to activity concentrations using camera- and reconstruction-specific calibration factors derived through phantom measurements, consistent with established methods (14).

### Compartment Model for Nonspecific and Specific Binding in Red Marrow

A pharmacokinetic compartment model was used to evaluate the nonspecific and specific activity distributions and retention in the red marrow regions. The blood activity concentration was determined from the descending aorta (Eq. 1). The blood activity concentration was assumed to be in equilibrium with the nonspecific activity concentration in the bone marrow cavity. The nonspecific activity concentration supplied the specific uptake in the red marrow compartments of the T9–L5 vertebrae or hip bones. The blood activity concentration was fitted to a biexponential function using trust-region–based nonlinear curve fitting in MATLAB (MathWorks):

$$C_{\text{blood}}(t)=A_{1}e^{-{\text{λ}}_{1}t}+A_{2}e^{-{\text{λ}}_{2}t},$$ Eq. 1

where *C*_blood_ is the blood activity concentration; *A*_1_, *A*_2_, λ_1_, and λ_2_ are the fit parameters; and *t* is the time after injection.

The specific activity concentration uptake and release rate in red marrow were determined by the compartment model in Equation 2:

$$\frac{dC_{\text{specific}}(t)}{dt}=k_{1}\cdot f\cdot C_{\text{blood}}(t)\;-\;k_{2}\cdot C_{\text{specific}}\left(t\right),$$ Eq. 2

where *C*_specific_ is the specific activity concentration, *k*_1_ and *k*_2_ are rate constants, and *f* is the fraction constant that adjusts the blood concentration to the nonspecific binding concentration obtained for the plasma volume distribution within the red marrow. The differential equation was solved numerically with a grid search to minimize the root mean square error between the determined activity concentration from the red marrow compartment in the SPECT images and the sum of the specific and nonspecific binding activity concentrations. Effective half-lives for the specific uptake in red marrow and tumors were estimated using an exponential fit to the later time points (>48 h) of the specific activity concentration.

### Red Marrow Dosimetry

The male and female skeletal dosimetry models from the University of Florida were used to estimate the absorbed dose to the red marrow for the vertebrae and hip bones (15,16). For the red marrow compartments, with the blood contribution removed, all activity was assumed to be located explicitly in the red marrow. To fit the skeletal dosimetry model in Table 2, red marrow activity concentrations were scaled with the corresponding red marrow volume fraction and red marrow volume to produce the time-integrated activity of the specific uptake of skeletal site *x* (Eq. 3). The cellularity in Table 2 is also tied to the skeletal dosimetry model and is the fraction of red marrow at a particular skeletal site that is hematopoietically active (red), which differs from the red marrow volume fraction, which also considers the amount of trabecular bone.

$${\text{TIA}}_{\text{RM},x}^{\text{specific}}=\underset{t}{\sum }\frac{C_{\text{specific},x}(t)}{f_{\text{RM},x}}\;V_{\text{RM},x},$$ Eq. 3

where *f*_RM,_*_x_* is the red marrow volume fraction, *V*_RM,_*_x_* is the red marrow volume, and ${\text{TIA}}_{\text{RM},x}^{\text{specific}}$is the time-integrated activity of the specific uptake. The contribution from blood-based irradiation (i.e., the nonspecific uptake) was accounted for by assuming equivalence between the activity concentration in the blood and red marrow and was scaled in a similar manner at the respective skeletal site. The absorbed dose to the red marrow was estimated using S values (Table 2) for a particular skeletal site from our previous study and the sum of specific and nonspecific time-integrated activity (Eq. 4) (17).

$$D_{\text{RM},x}=\left({\text{TIA}}_{\text{RM,}x}^{\text{specific}}+{\text{TIA}}_{\text{blood,}x}^{\text{nonspecific}}\right)\;S_{x}(\text{RM}\leftarrow \text{RM}),$$ Eq. 4

where $D_{\text{RM},x}$is absorbed dose to the red marrow. A blood-based approach with the activity concentration in blood equal to that in the red marrow, as suggested by the European Association of Nuclear Medicine guidelines, was performed to estimate the average red marrow absorbed dose to the whole body by scaling the nonspecific activity concentration from the aorta with the total amount of red marrow (Table 2) and using the skeletal averaged S values (Eq. 5) (18):

$$D_{\text{RM}}^{\text{blood-based}}=\;S_{\text{skeletal}\;\text{average}}\left(\text{RM}\leftarrow \text{RM}\right)\underset{t}{\sum }\frac{C_{\text{blood}}(t)}{f_{\text{RM}}}\;V_{\text{RM}}.$$ Eq. 5

**TABLE 2.** ^177^Lu S Values and Volumes and Volume Fractions of Red Marrow from University of Florida Male/Female Hybrid Phantom at Skeletal Sites Used for Absorbed Dose Calculations in This Study

Skeletal site	S (RM ← RM) (mGy/MBq/s)		Red marrow volume (cm^3^)		Red marrow volume fraction		ICRP 70 cellularity (%)
	Male	Female	Male	Female	Male	Female	
T vertebrae	1.05E−04	1.35E−04	147.2	112.5	0.63	0.62	70
L vertebrae	1.05E−04	1.08E−04	146.9	141.0	0.63	0.61	70
T/L vertebrae	5.25E−05	6.00E−05	294.0	253.5	0.63	0.62	70
Hip bones	3.85E−05	5.26E−05	299.7	225.5	0.43	0.46	48
Skeletal average	1.07E−05	1.40E−05	1170	904	0.52	0.47	—

ICRP = International Commission on Radiological Protection.

## RESULTS

Compartment modeling demonstrated an initial specific uptake and slow release phase of [^177^Lu]Lu-DOTATATE for all patients and skeletal sites studied. One patient (patient 5) had metastases in all vertebrae and the hip bones and was removed from all calculated averages in this study.

**FIGURE 2.**
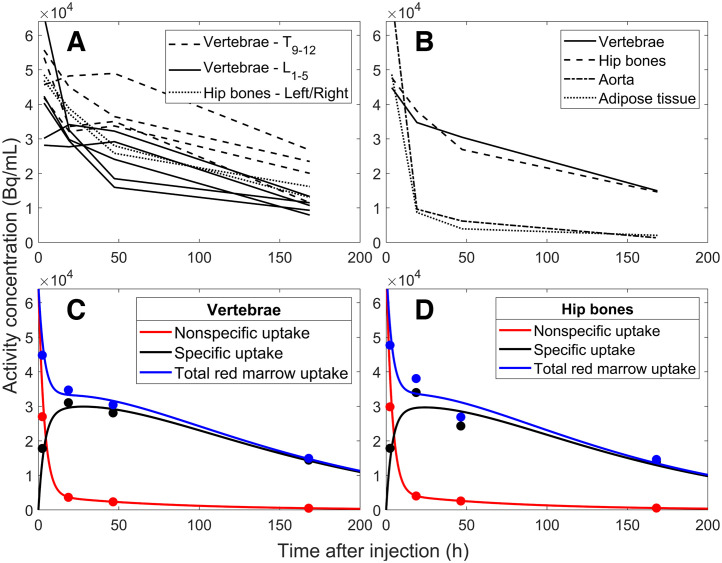
Biodistributions of 70-y-old woman (patient 1) with liver metastases. Activity concentrations from delineated red marrow regions in SPECT images are shown before (A) and after (B) averaging. B also presents biodistributions for aorta and adipose tissue regions. C and D show results from compartment model, which is based on data shown in B, separating specific and nonspecific uptake for mean of T9–L5 vertebrae (C) and mean of left and right hip bones (D).

Figure 2 shows the results for a 70-y-old woman (patient 1) with liver metastases. Figure 2A shows differences in uptake between the 9 delineated vertebrae as well as the left and right hip bones, which were averaged (Fig. 2B) for all vertebrae and both hip bones, respectively, before use in the compartment model. Figure 2B highlights the characteristic contrasting biodistributions of the skeletal sites compared with the aorta and subcutaneous adipose tissue, showing longer elimination times for the red marrow in the vertebrae and hip bones. The total self-absorbed dose to the red marrow was estimated to be 0.093 Gy/GBq in the vertebrae (Fig. 2C) and 0.085 Gy/GBq in the hip bones (Fig. 2D). The contribution to the total self-absorbed dose from the specific uptake in the red marrow in the vertebrae and hip bones was 89% and 93%, respectively, and that from the nonspecific uptake in the blood compartments was 11% and 7%. As a result of the small volume of the bone cavity and subsequent statistical insufficiencies in the VOI, data collected for the sternum were not evaluated.

### Overall Biodistributions in Patients With and Without Metastases

**FIGURE 3.**
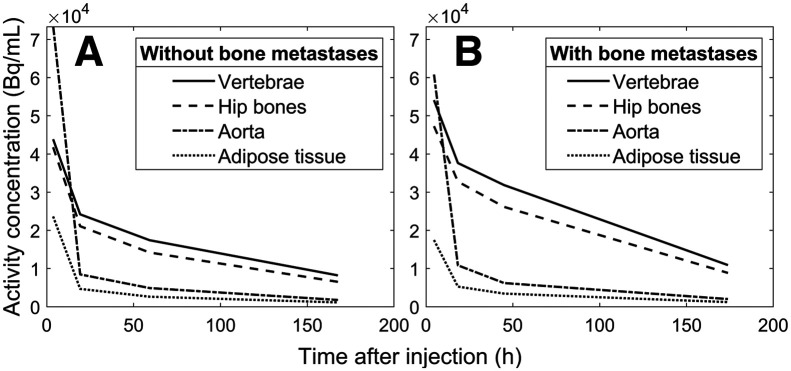
Mean values for biodistributions at 4 time points in T9–L5 vertebrae, hip bones, aorta, and adipose compartments from SPECT images of 10 patients without bone metastases (A) and 6 patients with confirmed bone metastases (patient 5 was removed) (B).

In the 10 patients without confirmed bone metastases, the activity concentrations in the delineated T and L vertebrae and hip bones showed a rapid initial distribution phase similar to that of the descending thoracic aorta compartment, followed by late elimination (Fig. 3A). After the initial rapid distribution, all skeletal sites had activity concentrations that were higher than the activity concentration in the blood. In contrast, the low uptake in adipose tissue had similar biodistributions to blood. The 7 patients with confirmed bone metastases had higher activity concentrations in the skeletal sites than the patients without bone metastases (Fig. 3B). Biodistributions for each patient are provided in Supplemental Figures 1–17 (supplemental materials are available at http://jnm.snmjournals.org).

### The Mean Specific and Nonspecific Uptake of [^177^Lu]Lu-DOTATATE

The mean biodistributions obtained from the compartment model (Fig. 4) followed the trends observed in the SPECT images with retention in the red marrow compartments. The retention at a particular skeletal site is connected to the amount of red marrow, showing higher activity concentrations in the delineated vertebrae than in the hip bones. Specific and nonspecific biodistributions for each patient are presented in Supplemental Figures 1–17.

**FIGURE 4.**
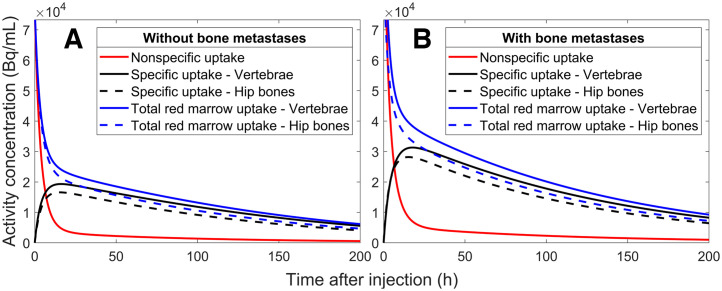
Mean compartment model biodistributions for aorta, T9–L5 vertebrae, and hip bones in all patients without (A) and with (B) bone metastases.

**FIGURE 5.**
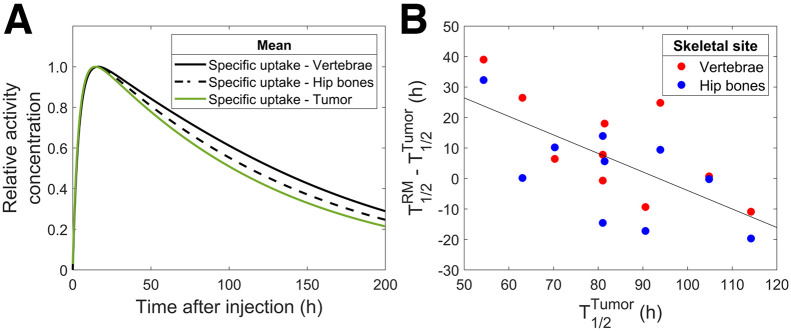
Mean relative biodistributions of vertebrae, hip bones, and tumors for 10 patients without bone metastases (A) and difference between effective half-life of ^177^Lu in red marrow sites and tumor vs. effective half-life of ^177^Lu in tumors (B).

To visually compare the biodistributions from specific uptake in the vertebrae, hip bones, and tumors for the 10 patients without bone metastases, activity concentrations were divided by their respective maxima (Fig. 5A). The mean effective half-life of the elimination phase was estimated to 94, 85, and 83 h for the vertebrae, hip bones, and tumors, respectively. The correlation of the difference between the elimination phases for tumor and red marrow and the elimination phase for tumor (Fig. 5B) was statistically significant (*P* = 0.0015). Individual biodistributions for the tumors are presented in Supplemental Figures 18–20.

### Absorbed Dose

**FIGURE 6.**
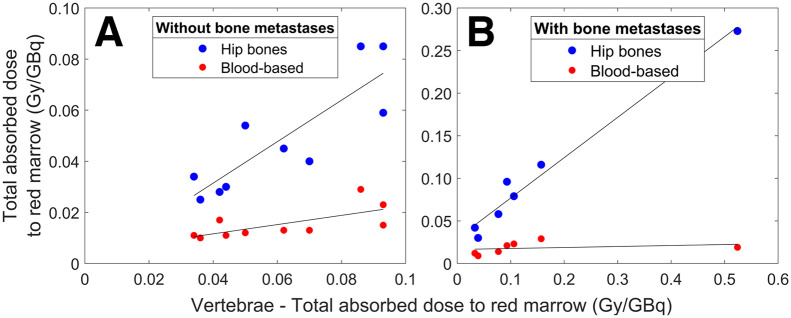
Total absorbed dose to red marrow (Gy/GBq) estimated for vertebrae compared with hip bones and blood-based methodology for 10 patients without bone metastases (A) and 7 patients with bone metastases (B).

Patient-specific absorbed dose was estimated for all 17 patients (Table 3). Using the T9–L5 vertebrae and combining the contributions from the specific and nonspecific uptake, the median (±SD) absorbed dose to the red marrow was 0.056 ± 0.023 Gy/GBq and 0.085 ± 0.046 Gy/GBq for patients without and with bone metastases, respectively. For the hip bones, the corresponding red marrow absorbed doses were 0.043 ± 0.022 Gy/GBq and 0.069 ± 0.033 Gy/GBq. The mean contribution to the total absorbed dose by specific uptake to the red marrow was 59% and 65% for the vertebrae and 62% and 69% for the hip bones in patients without and with bone metastases, respectively. Patient 5, who had bone metastases in all vertebrae and hip bones, had an estimated red marrow absorbed dose of 0.52 Gy/GBq and 0.27 Gy/GBq in the vertebrae and hip bones, respectively. The blood-based methods produced median absorbed red marrow doses of 0.013 ± 0.006 Gy/GBq and 0.017 ± 0.008 Gy/GBq in patients without and with bone metastases, respectively, 4.1 and 3.0 times lower than the median estimates in the vertebrae and hip bones (Fig. 6). Differences between the dosimetry methods and skeletal sites are visualized in Figure 6.

**TABLE 3.** Absorbed Dose to Red Marrow Estimated Using Image-Based Activity Concentrations in T9–L5 Vertebrae, Hip Bones, and Thoracic Aorta

Patient no.	Bone metastases	Vertebrae			Hip bones			Blood-based (skeletal average)
		Specific	Nonspecific	Total	Specific	Nonspecific	Total	
1	No	0.068	0.026	0.093	0.066	0.019	0.085	0.015
2	No	0.025	0.019	0.044	0.016	0.014	0.030	0.011
3	Yes	0.016	0.018	0.033	0.028	0.013	0.042	0.012
4	No	0.022	0.014	0.036	0.014	0.011	0.025	0.010
5*	Yes	0.496	0.028	0.524	0.251	0.022	0.273	0.019
6	No	0.018	0.016	0.034	0.022	0.012	0.034	0.011
7	No	0.020	0.023	0.042	0.012	0.017	0.028	0.017
8	No	0.029	0.021	0.050	0.039	0.015	0.054	0.012
9	No	0.038	0.024	0.062	0.028	0.017	0.045	0.013
10	No	0.047	0.023	0.070	0.024	0.017	0.040	0.013
11	Yes	0.065	0.041	0.106	0.049	0.030	0.079	0.023
12	Yes	0.052	0.025	0.077	0.040	0.018	0.058	0.014
13	No	0.052	0.041	0.093	0.030	0.030	0.059	0.023
14	Yes	0.056	0.037	0.093	0.069	0.027	0.096	0.021
15	Yes	0.114	0.042	0.157	0.084	0.032	0.116	0.029
16	Yes	0.026	0.013	0.039	0.020	0.010	0.030	0.009
17	No	0.043	0.043	0.086	0.052	0.033	0.085	0.029

* Removed from all averages because of large number of bone metastases.

Data are Gy/GBq. For vertebrae and hip bones, absorbed dose contributions were divided into specific (somatostatin receptor type 2–based) and nonspecific (blood-based, Eq. 4). Equation 5 was used for solely blood-based dosimetry in right column.

## DISCUSSION

In this study, the biodistribution of [^177^Lu]Lu-DOTATATE in red marrow cavities was investigated using SPECT/CT images. According to Oomen et al., somatostatin receptor type 2 is explicitly expressed by CD34-positive red marrow cells, most frequently on the CD117-positive fraction of CD34-positive cells. This subgroup comprises less than 1% of bone marrow cells and is found among pluripotent hematopoietic stem and progenitor cells (11,19). In line with this, we present evidence of specific uptake of [^177^Lu]Lu-DOTATATE in the red marrow regions of the T9–L5 vertebrae and hip bones in 17 patients, highlighting discrepancies between image-based and blood-based dosimetry methods. Blood-based methods fail to account for the specific red marrow uptake and, therefore, produce inadequate estimates of the absorbed dose to red marrow (2). In general, hematopoietic CD34-positive cells are associated with high proliferation capabilities, as well as cell differentiation, and are particularly important in hematopoiesis (20). When the duration of hematologic toxicities was studied in 203 patients after [^177^Lu]Lu-DOTATATE radiotherapy, a mean of 12 mo (range, 2–22 mo) was needed for blood count recovery, indicating the sensitivity and importance of the red marrow cells expressing somatostatin receptor type 2 (1).

### Dosimetry: Specific Uptake

Although uptake was visible in the red marrow compartments on the SPECT images (Figs. 2A, 2B, and 3; Supplemental Figs. 1–17), we attempted to quantify the distribution and retention in the red marrow by applying a compartment model to the activity concentrations from the aorta and vertebrae/hip bones. We chose a dosimetry approach using the S values from a previous publication and assumed that the uptake in the red marrow compartments provided by the compartment model was located exclusively in the red marrow. This leads to a higher absorbed dose to red marrow than does assuming evenly distributed uptake in the spongy bone but appears reasonable because our results show longer elimination times in the red marrow compartments than in the control compartments in all patients. To calculate the absorbed dose to red marrow in the vertebrae or hip bones, it is necessary to scale the activity concentrations with the corresponding reference red marrow volume fraction and volume. This represents a considerable assumption, as multiple studies have reported large interpatient variations in red marrow mass, particularly considering the therapeutic background of this patient group (21–23). However, red marrow volume can be estimated by MRI- or CT-based methods before therapy and would conceivably be a significant step forward in patient-specific red marrow dosimetry because the red marrow volume, at least hypothetically, should be connected to the observed red marrow response (24–26). For patients without bone metastases, our methods generated absorbed doses to the red marrow that are in agreement with previous reports (Table 1). The lower absorbed doses observed for the hip bones are a consequence of the slightly lower uptake (Figs. 2A, 2B, and 3; Supplemental Figs. 1–17), which in turn is due to the lower cellularity and larger volume of the hip bone cavities. Although the total extent of the bone metastases in these patients is unknown, a 2018 study of 677 patients with neuroendocrine neoplasms concluded that the vertebrae are the skeletal site most frequently associated with bone metastases, followed by the pelvic region (27). This can perhaps be observed through the smaller regression line slope for the hip bone than of the vertebrae in patients with bone metastases (Fig. 6), suggesting that the hip bone is more appropriate for red marrow dosimetry in patients with bone metastases.

### Dosimetry: Nonspecific Uptake

The blood-based contribution to the irradiation of red marrow was estimated by assuming an equivalence between the distribution of blood vessels and red marrow in the bone cavities, a premise that is partially supported by studies using healthy-bone-marrow biopsies that demonstrated that both CD34-positive cells and blood vessels in the spongy bone are distributed along negative spatial gradients from the trabecular bone outward (4,28). This enabled us to use the same S values for the blood-based irradiation and reduced the likelihood of underestimating that fraction of the absorbed dose. However, according to our results, the use of a solely blood-based approach should be avoided because it would, on average, lead to a 70% underestimation of the total absorbed red marrow dose due to differences between the biodistributions of the blood and red marrow. The generated median absorbed doses were close to the 0.016 Gy/GBq reported for the mainly blood-based dosimetry in 200 patients (29).

### Imaging

Quantitative SPECT imaging of the red marrow is challenging because of the low activity concentrations in the cavities, limited spatial resolution, and photon spill-in from neighboring regions (30). These difficulties can be partially addressed with Monte Carlo–based SPECT reconstructions (31). The result is a reconstructed image more accurately corrected for scattered photons. Increasing the number of iterations (updates) used in SPECT OSEM reconstructions will generally reduce image blur but increase image noise. Even if the Monte Carlo OSEM reconstructions performed in this study had an improved resolution and noise profile compared with conventional OSEM reconstructions, the presence of noise contributes to the variability of the imaged uptake in the vertebrae, making quantification in separate vertebrae challenging. Consequently, we used the mean activity concentration in the vertebrae and hip bones to obtain robust results. Image-based determination of activity concentrations in the aorta using PET rather than blood sampling has been proven accurate (13). The approach has been extended to SPECT imaging, particularly for measuring myocardial blood flow, and the method was deemed reasonable to use in our study (32,33).

### Limitations

Of the 17 patients included, 7 had confirmed bone metastases, making the absorbed dose estimates for some of these patients unreliable because the tumor uptake gives higher absorbed doses to the red marrow compartment and poses further questions about the health of the red marrow and the accuracy of the delineation of normal tissue. However, the inclusion of multiple skeletal sites, such as the hip bones or proximal humerus, in the absorbed dose estimates is a viable option for reasonable red marrow dose estimates, as patients with significant bone involvement in the vertebrae do not necessarily maintain increased uptake in the hip bones (34). Two patients (patients 3 and 7) had tumors in the lungs, which were observed through an uncharacteristic late elimination in the thoracic aorta possibly due to photon spill-in from these tumors. This is an effect of the limited resolution in our SPECT images and resulted in higher nonspecific absorbed dose contributions.

The uptake phase in tumor tissue and red marrow compartments was similar (Fig. 5A), suggesting that direct binding to somatostatin receptors is the main explanation for the observed specific uptake. However, a statistically significant slower elimination phase was observed for the red marrow activity in patients having tumors with a fast elimination phase than in those having tumors with slow elimination (Fig. 5B). This may indicate that transferrin, a highly effective iron transporter that can bind many metallic ions, is involved in bringing to the red marrow the TATE-free ^177^Lu released by the tumors. In vitro studies have also demonstrated direct metal ion transchelation from the DOTA chelator to the transferrin transporting site (35). Such transchelation might also occur for, for example, ^177^Lu-PSMA ligands, with a resulting increase in red marrow irradiation; however, further studies are needed.

## CONCLUSION

Using biodistributions from sequential SPECT images and multiple skeletal sites, we observed specific uptake in the studied red marrow compartments through late elimination, which we attribute to the presence of somatostatin receptor type 2 on CD34-positive hematopoietic stem cells in the red marrow. A compartment model was used to separate the blood-based and specific uptake contributions to the absorbed red marrow dose, implying that a purely blood-based dosimetry method systematically underestimates the absorbed dose with an amplitude highly patient-dependent. Furthermore, red marrow dosimetry at multiple skeletal sites is plausible and can be valuable for improved absorbed dose estimates in patients with bone metastases.

## DISCLOSURE

This work was supported by the Swedish Cancer Society, the King Gustav V Jubilee Clinic Cancer Research Foundation, the Swedish Research Council, and the Swedish State under an agreement between the Swedish government and the county councils: the ALF agreement. Peter Bernhardt is a cofounder of Theravision AB. No other potential conflict of interest relevant to this article was reported.

## Notes

KEY POINTS **QUESTION:** Can somatostatin-related uptake in the red marrow be observed consistently in SPECT images after [^177^Lu]Lu-DOTATATE treatment? **PERTINENT FINDINGS:** Specific uptake in the red marrow was observed in all 17 patients and skeletal sites, leading to an underestimation of the absorbed dose when using purely blood-based dosimetry methods. **IMPLICATIONS FOR PATIENT CARE:** Our findings support the statement that blood-based red marrow dosimetry should be avoided for somatostatin receptor type 2 radioligands and that more accurate patient dosimetry can be obtained with SPECT-based methodology to determine the specific and nonspecific red marrow contribution to the mean absorbed dose.
